# Tertiary lymphoid structures in urothelial carcinoma: bridging spatial architecture with therapeutic vulnerability in the post-BCG era

**DOI:** 10.3389/fimmu.2026.1853633

**Published:** 2026-09-08

**Authors:** Xin Wei, Xin Pei, Shanshan Yu

**Affiliations:** 1 Department of Urology, China-Japan Union Hospital, Jilin University, Changchun, China; 2 Department of Anaesthesiology, China-Japan Union Hospital, Jilin University, Changchun, China

**Keywords:** BCG, bladder cancer, immunotherapy, spatial transcriptomics, tertiary lymphoid structures, tumor microenvironment

## Abstract

Urothelial carcinoma (UC) remains a persistent clinical challenge in the post-Bacillus Calmette-Guérin (BCG) era, particularly because a substantial proportion of patients with high-risk non-muscle-invasive bladder cancer (NMIBC) experience recurrence or progression despite standard intravesical immunotherapy. Although immune checkpoint inhibitors (ICIs) and antibody-drug conjugates (ADCs) have expanded the therapeutic armamentarium, widely studied biomarkers such as tumor mutational burden (TMB) and PD-L1 expression show limited and inconsistent predictive performance in UC, which may partly reflect their inability to capture the spatial organization and functional states of the tumor immune microenvironment (TIME). Tertiary lymphoid structures (TLS), ectopic lymphoid aggregates that develop in non-lymphoid tissues at sites of chronic inflammation or cancer, have emerged as candidate mediators and spatial markers of local adaptive immunity. In UC, TLS appear to exhibit spatial heterogeneity and may exist across a maturation continuum from early, disorganized lymphoid aggregates to mature, germinal center-containing follicles; their localization within the lamina propria, tumor stroma, or invasive front may be associated with distinct immune contexts and clinical outcomes. This review provides a urology-centric perspective on TLS biology in UC, emphasizing how the anatomical and immunological niche of the bladder, including urinary exposure, the urinary microbiome, BCG-associated inflammation, and stromal remodeling, may shape the conditions under which TLS develop and function. We critically discuss TLS maturation status as a potential spatial biomarker, while emphasizing that its relationship with BCG failure and subsequent response to PD-1/PD-L1 blockade or enfortumab vedotin (EV)-based therapy remains largely correlative and requires prospective validation. Furthermore, we explore emerging strategies that may modulate TLS-associated immune architecture, including STING agonist instillation and LTβR pathway modulation, and discuss how TLS could be tested as an adjunctive biomarker in future bladder-preservation studies for high-risk T1 disease. By synthesizing current evidence and methodological advances in spatial transcriptomics, we underscore the translational imperative of viewing UC as a malignancy with heterogeneous local immune architecture, including candidate tertiary lymphoid structures. At present, TLS should not be used as a stand-alone biomarker for selecting bladder preservation, radical cystectomy, intravesical therapy continuation, or systemic treatment switching.

## 1 Introduction: the unmet need beyond BCG failure

### 1.1 The therapeutic ceiling of intravesical BCG in high-risk NMIBC

Bacillus Calmette-Guérin (BCG) intravesical instillation has been used as the standard immunotherapy for non-muscle-invasive bladder cancer (NMIBC) for nearly half a century. BCG mediates antitumor activity by activating local innate and adaptive immune responses and is widely regarded as an early example of successful cancer immunotherapy in solid tumors (1). However, this classic therapy has a clear efficacy ‘ceiling’. Although BCG reduces recurrence risk, clinically important recurrence and progression continue to occur despite induction and maintenance treatment (2, 3). In high-grade T1 disease, this residual risk creates difficult clinical decisions regarding repeat TURBT, continued intravesical therapy, early radical cystectomy (RC), and timely transition to systemic or investigational bladder-sparing approaches.

### 1.2 From molecular biomarkers to spatial immunity: why TMB and PD-L1 only partially predict outcomes in bladder cancer

With the application of immune checkpoint inhibitors (ICIs) in advanced urothelial carcinoma, biomarkers such as tumor mutation burden (TMB) and PD-L1 combined positive score (CPS) have been extensively explored. However, in bladder cancer, the predictive value of these ‘systemic’ or ‘average’ level markers is limited and inconsistent. When used in isolation, TMB and PD-L1 expression provide incomplete predictive information for ICI response in UC, and their performance varies across cohorts, assays, treatment settings, and scoring systems (4). This limited predictive performance may partly reflect the marked heterogeneity of the tumor immune microenvironment (TIME). Traditional molecular biomarkers fail to capture the spatial distribution, interactions and functional states of immune cells within the tumor, yet these ‘spatial architecture’ characteristics are precisely what determine the efficacy of the immune response. For example, even with high PD-L1 expression, immunotherapy will remain ineffective if cytotoxic T cells are excluded from the tumor core or are in a state of exhaustion. Consequently, there is an urgent need for novel biomarkers that transcend single-dimensional approaches and can reflect the spatial organizational complexity of the TIME.

### 1.3 Introduction of the TLS concept: ‘organized aggregation’ of immune cells at the tumor margins and within the tumor - a special pathological structure from a urological perspective

Against this backdrop, Tertiary Lymphoid Structures (TLS) have been introduced into the field of bladder cancer research as an emerging concept of ‘spatial immunity’. TLS are ectopic lymphoid aggregates that form *de novo* at sites of chronic inflammation or cancer and may range from loose B- and T-cell aggregates to highly organized follicles containing B-cell zones, T-cell areas, follicular dendritic cell networks, germinal center-like reactions, and high endothelial venules (5). In urothelial carcinoma, TLS have been described in the tumor stroma, lamina propria, and invasive front, where they may contribute to local adaptive antitumor immune responses (6–12). From a urological pathology perspective, TLS is not merely a simple lymphocytic infiltration, but a distinct pathological structure with a specific spatial architecture. In retrospective UC cohorts, TLS presence, density, maturation state, and spatial distribution have been associated with prognosis or treatment-related immune context; however, TLS has not been prospectively validated as a stand-alone predictive biomarker in bladder cancer (6–9). Consequently, TLS provides a spatial dimension for understanding immune heterogeneity in bladder cancer and for generating testable therapeutic hypotheses.

### 1.4 Literature scope and evidence appraisal

This review was informed by targeted PubMed searches through 21 August 2026. Search concepts included tertiary lymphoid structures, B cells, CXCL13, BCG, urothelial carcinoma, bladder cancer, UTUC, PD-1/PD-L1, enfortumab vedotin, STING, LTbetaR, and spatial transcriptomics. English-language full original research articles and pivotal clinical trial reports were prioritized for mechanistic, pathological, prognostic, therapeutic, and clinical statements. Web of Science Core Collection was used for supplementary bibliographic cross-checking where institutional access was available; final source verification in this revision relied principally on PubMed and DOI/publisher metadata. Reviews were retained only for historical or general background, including explicitly labeled evidence from other tumor types.

## 2 The unique niche: anatomical and immunological prerequisites of TLS formation in urothelium

The formation of tertiary lymphoid structures (TLS) in urothelial carcinoma of the bladder is not a random occurrence, but rather is shaped by the unique anatomical and immunological microenvironment of the urinary tract. This ‘niche’ determines the developmental pattern, functional state and ultimate clinical significance of the TLS.

### 2.1 Urothelial mucosal exposure, urinary flow, and microbial signals as potential regulators of B-cell homing

As an organ for urine storage, the inner lining of the bladder—the urothelium—is continuously exposed to urine, a dynamic fluid environment rich in metabolic products. These unique physiological conditions constitute a distinctive “mucosal firewall”. On the one hand, continuous urinary flushing acts as a physical barrier that may limit pathogen colonization, yet simultaneously poses a challenge to the stable residence of immune cells. On the other hand, urine is not sterile; it harbors a complex microbial community, namely the urinary tract microbiome (13). Commensal or pathogenic bacteria and their metabolites, including short-chain fatty acids and lipopolysaccharides, may engage pattern-recognition pathways in uroepithelial and lamina propria immune cells, thereby contributing to local chemokine and cytokine milieus under inflammatory or tumor-associated conditions (14). CXCL13, through its interaction with CXCR5 on B cells, is a key B-cell chemoattractant and one component of the chemokine network involved in B-cell recruitment and lymphoid organization (15). Therefore, rather than being viewed as a direct or inevitable consequence of urinary microbial stimulation, TLS formation in the bladder should be interpreted as a candidate spatial immune process that may arise under the combined influence of urinary exposure, microbial or inflammatory signals, stromal remodeling, and tumor antigenic stimulation.

### 2.2 BCG-associated “scar immunity” as a conceptual model for post-treatment stromal remodeling

Bacillus Calmette-Guerin (BCG) bladder instillation is a classic immunotherapy for the treatment of non-muscle-invasive bladder cancer (NMIBC), and its effects extend beyond acute inflammatory responses. Repeated BCG instillation may leave a chronic inflammatory and stromal-remodeling imprint in the bladder lamina propria, which we refer to in this review as ‘scar immunity’. This term is used as a conceptual description of a post-treatment immune-stromal state, rather than as an established pathological entity. BCG has a well-established local inflammatory immunotherapy rationale (general clinical background) (1, 16). A proposed mechanism is that prolonged immune stimulation may alter the local stromal microenvironment: activated fibroblasts or stromal cells may acquire lymphoid tissue-organizer-like features and upregulate homing molecules such as CXCL13, CCL19, and CCL21, which are involved in lymphoid aggregation and organization. This proposed stromal program is derived from general TLS biology and has not been demonstrated in paired longitudinal post-BCG human UC specimens. The cellular sources, timing, and functional relevance of CXCL13, CCL19, and CCL21 in this setting remain unknown; extracellular matrix remodeling may provide a physical scaffold for immune-cell clustering. The available UC pathology literature does not establish that BCG induces *de novo* TLS or that a TLS-rich microenvironment predicts BCG benefit. These observations therefore support only a testable hypothesis: BCG-associated inflammation may create cytokine and stromal conditions permissive for TLS formation or maturation in some tumors, but this model requires prospective validation.

#### 2.2.1 Canonical TLS biology and evidence boundaries

Canonical TLS biology (evidence from other tumor types and non-malignant chronic inflammatory settings) provides a framework for interpreting these observations. Lymphoid-tissue-inducer-like programs and related immune signals may activate the LTalpha1beta2-LTbetaR axis, thereby promoting stromal organizing programs. Local fibroblasts may acquire follicular-dendritic-cell-like or fibroblastic-reticular-cell-like features and participate in chemokine networks involving CXCL13, CCL19, and CCL21. Endothelial cells may acquire HEV-like features that support lymphocyte entry, while mature germinal-center-like reactions depend on coordinated T follicular helper-cell-B-cell interactions, including MHC class II-T-cell receptor, CD40-CD40L, and ICOS-ICOSL signaling. PD-1 expression within this context should not be interpreted as evidence of terminal T-cell exhaustion by itself. These canonical mechanisms have not been demonstrated in paired longitudinal post-BCG human UC specimens; their cellular sources, temporal sequence, and functional relevance in UC remain to be validated (5, 17–22).

### 2.3 High-endothelial venules in urothelial carcinoma: clinicopathological associations and evidence boundaries

High endothelial venules (HEVs) are specialized postcapillary venules that facilitate lymphocyte trafficking into secondary lymphoid organs and, in some settings, into tertiary lymphoid structures (23–25). In urothelial carcinoma, histological studies have described associations between HEV-containing regions and TLS-associated immune architecture (6). These observations support the use of PNAd as a candidate architectural marker when evaluating TLS maturity. However, they do not establish HEV density as a measure of vascular adequacy, antitumor competence, immunotherapy sensitivity, or clinical outcome in individual patients. Prospective studies integrating HEV assessment with standardized TLS scoring and treatment-specific outcomes are required (7–9).

## 3 Heterogeneity of TLS in urothelial carcinoma: a spatial continuum

Tertiary lymphoid structures (TLS) are heterogeneous immune aggregates. In urothelial carcinoma (UC), their maturity, cellular composition, and spatial localization may be associated with distinct immune contexts and clinical outcomes (7–9). However, these histological features do not by themselves establish functional activity, treatment responsiveness, or prognosis in individual patients. Their clinical interpretation remains dependent on standardized definitions and prospective validation.

### 3.1 Descriptive histological assessment of TLS-like structures in bladder cancer

TLS-like structures in bladder cancer can be described along a histological continuum ranging from loose lymphoid aggregates to organized follicles with follicular dendritic cell networks, germinal center-like activity, and HEV-like vessels (6–9). This framework is descriptive rather than universally standardized, and morphology alone should not be interpreted as a direct measure of antitumor function, BCG responsiveness, or clinical trajectory.

Early TLS/lymphoid aggregates: These structures are characterized by loose aggregates of CD20-positive B cells and CD3-positive T cells without clear follicular organization, follicular dendritic cell networks, or germinal center-like features (6–9). Their morphology indicates local immune-cell recruitment but does not establish functional incompetence, immune suppression, or a specific prognosis in UC. Their clinical significance remains uncertain and requires evaluation using standardized spatial and longitudinal approaches (6–9).

**Figure 1:**
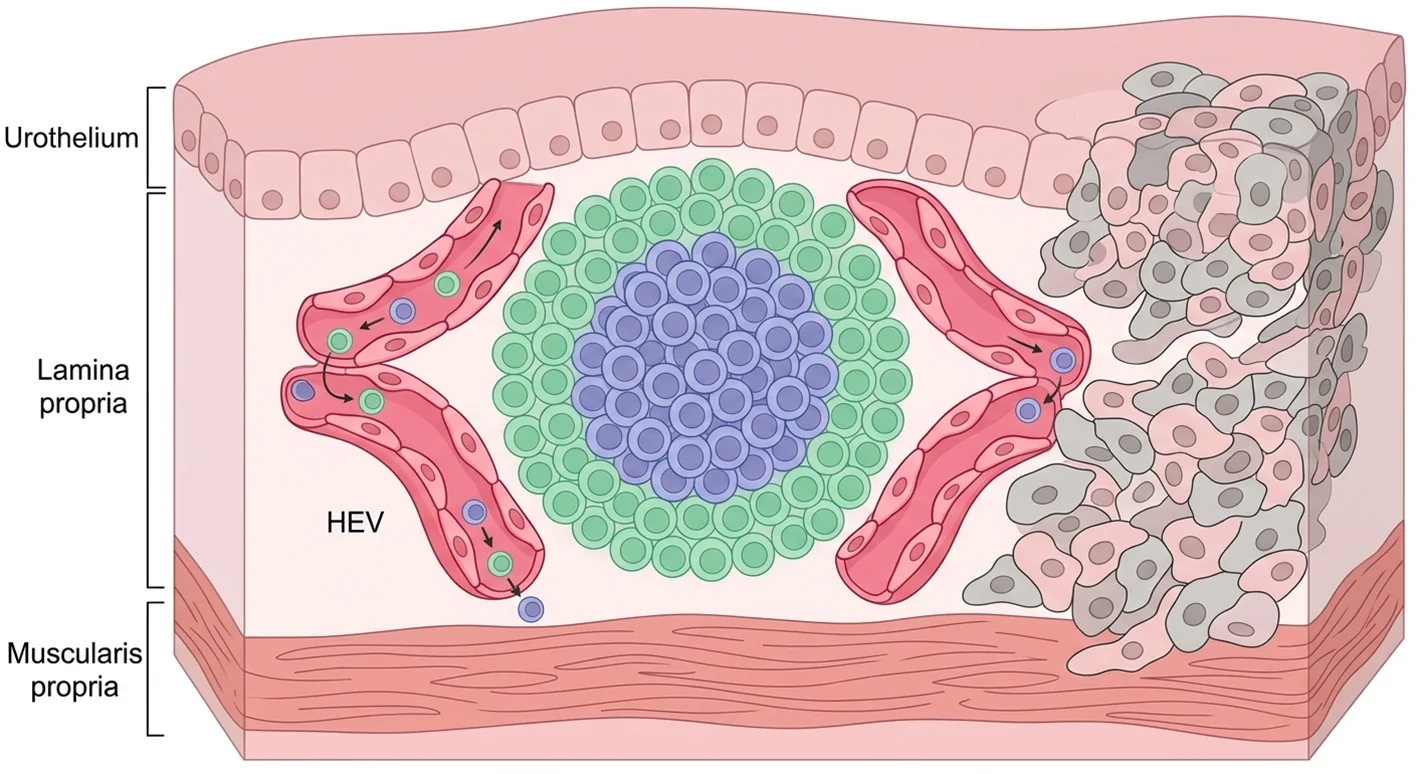
Spatial architecture of tertiary lymphoid structures (TLS) in urothelial carcinoma of the bladder. Schematic representation of the bladder wall depicting the spatial relationship between TLS and key anatomical layers. A mature TLS is illustrated within the lamina propria, characterized by a dense cluster of CD20+ B cells (blue) forming a follicular structure surrounded by a T-cell zone enriched in CD8+ cytotoxic T cells (green). High endothelial venules (HEV, pink) are shown adjacent to the TLS, serving as specialized portals for continuous lymphocyte recruitment from the peripheral blood. The overlying urothelium, underlying muscularis propria, and invasive tumor cell nests (grey) are indicated to highlight the proximity of TLS to the invasive front. This schematic illustrates a proposed spatial relationship between TLS-like structures and bladder-wall compartments. It is intended as an anatomical and histological representation and does not establish that TLS at a given location functions as an immune hub or mediates a specific clinical outcome.

Mature TLS: Mature TLS are morphologically characterized by organized B-cell follicles, T-cell areas, follicular dendritic cell networks, germinal center-like activity, and, in some cases, PNAd-positive HEV-like vessels (6–9). The spatial organization of a mature TLS within the bladder wall, including its relationship with the urothelium, lamina propria, HEVs, and invasive tumor front, is schematically illustrated in **Figure 1**. In retrospective UC cohorts, mature TLS-associated immune features have been associated with immune-active microenvironments and favorable clinical outcomes (6–9). However, these associations do not demonstrate that mature TLS directly mediates BCG response, establishes antitumor immune memory, or predicts benefit from a specific treatment in individual patients.

Regulatory or inhibitory-state immune-cell enrichment within TLS-like aggregates: Some TLS-like aggregates in UC may contain increased FOXP3-positive regulatory T cells or immune cells expressing PD-1, LAG-3, or TIM-3 (7–9). This is a descriptive and non-standardized immune contexture rather than an established TLS subtype. Expression of these markers alone does not establish terminal T-cell exhaustion, immune tolerance, BCG failure, disease progression, or resistance to immunotherapy. Their interpretation requires lineage-resolved multiplex spatial analysis and longitudinal clinical validation.

Recent clinicopathological studies in bladder urothelial carcinoma have further highlighted that TLS are heterogeneous structures with distinct maturation states and prognostic associations. To improve practical readability and to avoid overinterpreting TLS morphology, **Table 1** summarizes candidate TLS states, histological features, markers, locations, proposed clinical implications, and current evidence levels in UC (7–9).

**Table 1:** Candidate TLS states, histological features, markers, locations, and clinical implications in urothelial carcinoma.

TLS subtype/status	Key histological features	Candidate markers	Common location in UC	Proposed clinical implication	Evidence level
Early TLS/lymphoid aggregate	Loose B- and T-cell aggregates without clear follicular organization	CD20, CD3; absent or weak CD21/CD23	Lamina propria, tumor stroma, invasive front	May indicate immune recruitment, but functional competence is uncertain	Observational; definitions vary
Mature TLS	Organized B-cell follicle, T-cell zone, follicular dendritic cell network, germinal center-like reaction, and HEVs	CD20, CD3, CD21/CD23, BCL6, Ki67, PNAd	Lamina propria, tumor stroma, invasive margin	Associated with immune-active microenvironments and favorable outcomes in retrospective studies; candidate research marker, but predictive value for BCG or ICI response remains unvalidated	Retrospective and correlative
Regulatory or inhibitory-state immune-cell enrichment within TLS-like aggregates	Increased FOXP3-positive cells or expression of PD-1, LAG-3, or TIM-3 within a lymphoid aggregate; morphology and marker expression do not define a discrete TLS subtype.	FOXP3, PD-1, LAG-3, TIM-3; interpretation requires cell-lineage and spatial context.	Not established.	No independent prognostic or predictive implication has been established in UC.	Hypothesis-generating (7–9).
TLS-absent/immune-cold phenotype	Lack of organized lymphoid aggregates; sparse immune infiltration	Low CD20/CD3/CXCL13/PNAd signal	Variable	May indicate limited immune recruitment; should be interpreted together with established clinicopathological risk factors	Indirect evidence

### 3.2 Spatial localization and immune contexture: observational associations and unresolved functional significance

The spatial distribution of TLS within tumor tissue is not random, but is closely linked to their functional specialization and clinical significance, providing a profound insight into the dynamic geography of tumor-immune interactions.

In NMIBC, lymphoid aggregates or TLS-like structures have been reported in the lamina propria adjacent to the urothelium (8, 9). Their superficial location may place them in proximity to intravesical BCG exposure and mucosal immune-cell trafficking. However, no longitudinal human study has established that superficial TLS are the primary site of BCG antigen capture, antigen presentation, durable local memory formation, or recurrence prevention. These proposed functions are hypothesis-generating and require paired longitudinal post-BCG tissue studies.

In MIBC, TLS-like structures have been described in tumor stroma and at the invasive margin in selected cohorts (6–9). Their spatial distribution may reflect local interactions among tumor cells, stromal cells, vasculature, and infiltrating immune cells. However, location alone does not define TLS maturity, T-cell exhaustion, immune escape, or tumor aggressiveness. In particular, no validated evidence shows that deep or invasive-front TLS enriched in regulatory or inhibitory-marker-positive cells independently predict progression from T1 to T2 disease. Such associations should be tested prospectively together with established pathological risk factors (7–9).

Practical assessment of TLS maturity in TURBT specimens: For clinical translation, TLS assessment must be feasible in TURBT specimens. A pragmatic research workflow could begin with hematoxylin and eosin screening for lymphoid aggregates, followed by a limited IHC panel. CD20 and CD3 can confirm B- and T-cell compartmentalization; CD21 or CD23 can identify follicular dendritic cell networks; BCL6 and/or Ki67 can support germinal center activity; and PNAd can evaluate HEV formation. When clinically or scientifically relevant, FOXP3, PD-1, LAG-3, and TIM-3 may help identify regulatory or exhausted immune-cell enrichment. Potential assessment time points include baseline TURBT, repeat TURBT, post-induction BCG biopsy/TURBT, and recurrence specimens. This workflow remains investigational and requires prospective validation before TLS maturity can guide TURBT, intravesical therapy, systemic treatment, or radical cystectomy.

## 4 TLS in the post-BCG setting: clinical context and evidence gaps

In the post-BCG setting, particularly in patients with BCG-unresponsive high-risk NMIBC, clinical management increasingly involves difficult choices among radical cystectomy, alternative intravesical approaches, systemic immunotherapy, clinical trials, and emerging bladder-sparing combinations (1). TLS-associated immune architecture has emerged as a candidate biological feature for investigating treatment-associated immune states in this setting. However, TLS status has not been prospectively validated as a predictive biomarker of BCG response or subsequent treatment benefit (8, 9, 26).

### 4.1 A re-examination of TLS status in BCG-unresponsive disease

Direct evidence comparing TLS maturity, spatial distribution, or cellular composition between BCG-refractory and BCG-recurrent tumors is currently limited. Retrospective UC studies have reported associations between TLS-related immune features and outcomes in selected cohorts (6–9); however, these data do not establish a reproducible TLS phenotype for either clinical category. The BCG-treated NMIBC study by Drachneris et al. evaluated recurrence-associated immune-cell distributions, but it did not define a longitudinal TLS phenotype (26). Whether pre-existing or post-treatment TLS architecture differs between BCG-responsive, BCG-refractory, and BCG-recurrent disease remains an important prospective research question.

**Figure 2:**
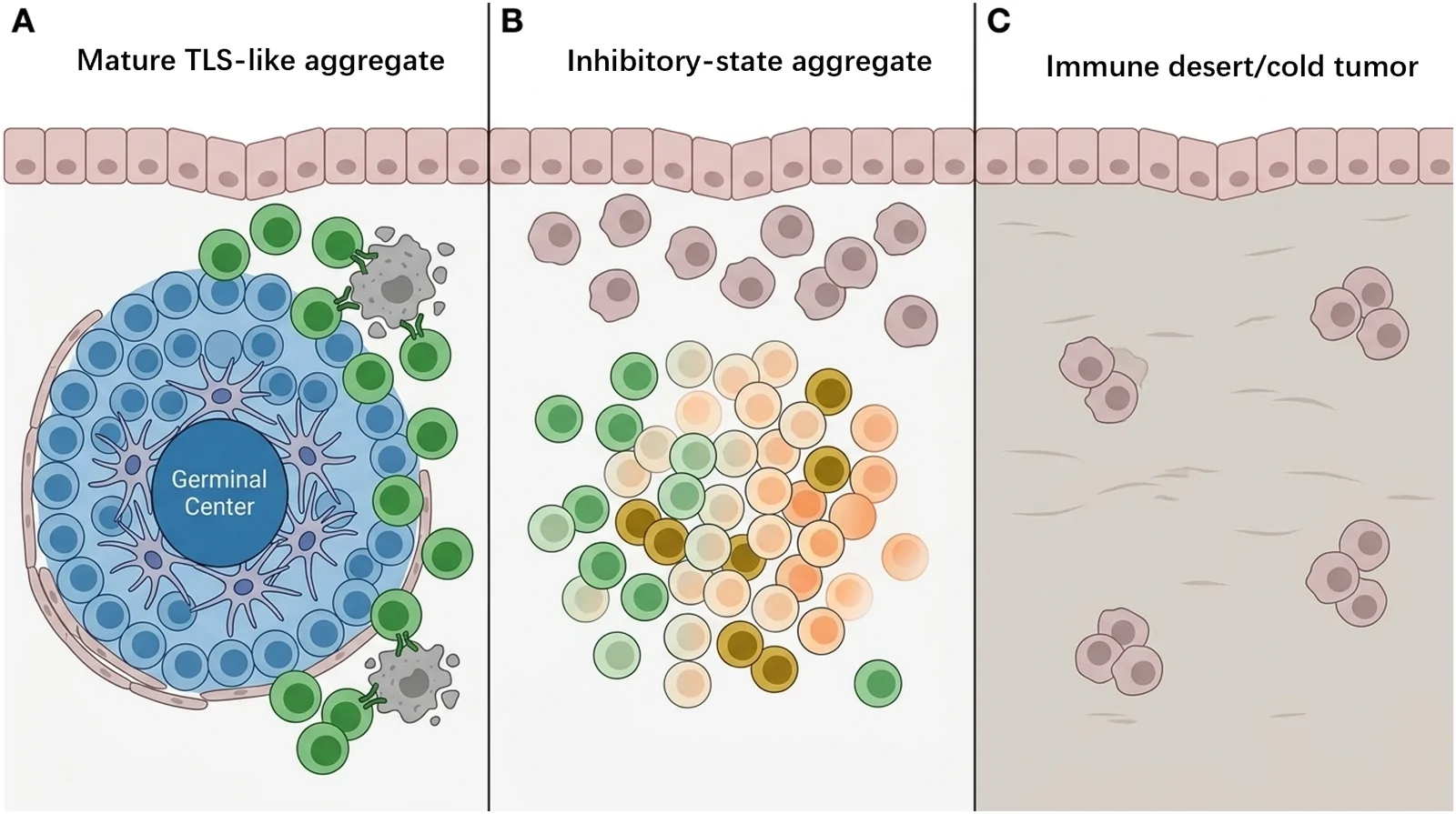
Hypothesis-generating “trifurcation” model of TLS-associated immune trajectories after intravesical BCG. This conceptual schematic proposes three possible, non-mutually exclusive immune outcomes after repeated BCG exposure: **(A)** maturation of organized TLS with germinal center-like features and HEV-like features, potentially supporting antitumor immunity; **(B)** development or persistence of TLS-like aggregates with regulatory or inhibitory-state immune-cell enrichment, a descriptive and non-standardized pattern that may coexist with immune escape; and **(C)** failure of TLS neogenesis, resulting in an immune-cold tumor microenvironment. This model has not been prospectively validated in longitudinal human TURBT cohorts and should not be used as a clinical classification system.

Repeated BCG exposure is associated with local treatment-related inflammation (general clinical background) (1, 16). Whether such changes generate *de novo* TLS, alter the maturation of pre-existing TLS, or simply coexist with treatment-related inflammation remains unknown; the available UC studies do not resolve this longitudinal question (8, 9, 26). Similarly, the presence of FOXP3-positive cells or PD-1, LAG-3, and TIM-3 expression within post-treatment lymphoid aggregates does not establish an exhausted TLS subtype or a mechanism of BCG failure. These issues require paired longitudinal TURBT cohorts with multiplex spatial profiling and treatment-specific outcome data. **Figure 2** therefore presents a conceptual and hypothesis-generating research framework rather than a biological or clinical classification system.

### 4.2 TLS maturity and PD-1/PD-L1 blockade: clinical relevance and unresolved predictive value

The presence of TLS has been associated with ICI response in several cancers and in some UC datasets, but its predictive value in routine UC treatment selection remains unvalidated.

Across tumor types, mature TLS are associated with B-cell differentiation, germinal center-like activity, and plasma-cell accumulation (5, 17, 19–21). In UC, selected retrospective datasets have reported associations between B-cell or TLS-related immune features and clinical outcomes (7–9). However, these associations do not demonstrate that intratumoral antibodies mediate antibody-dependent cellular cytotoxicity, that TLS directly causes checkpoint-inhibitor benefit, or that mature TLS can identify individual patients who will respond to PD-1/PD-L1 blockade. The contribution of T follicular helper-cell-B-cell interactions and humoral immunity to ICI response in UC remains to be defined in prospective translational studies.

Interpretation of inhibitory immune-cell markers within TLS-like aggregates: Lymphoid aggregates lacking germinal center-like features should not be assumed to be functionally ineffective or intrinsically immunosuppressive on the basis of morphology alone (6–9). In general TLS biology, CXCL13 contributes to lymphocyte positioning and lymphoid organization, but its expression is not specific for a particular TLS state (15, 27). In a neoadjuvant PD-1 blockade study of muscle-invasive bladder cancer, Escherichia coli-specific CXCL13-producing T follicular helper cells were associated with clinical efficacy (28). This observation highlights a context-specific CXCL13-T follicular helper-cell program and does not validate an exhausted TLS subtype. Likewise, PD-1, LAG-3, TIM-3, or FOXP3 expression may indicate distinct activation, regulatory, or differentiation states depending on cell lineage and spatial context. Current UC evidence does not establish that CXCL13 preferentially recruits exhausted CD8-positive T cells into immature TLS, or that such aggregates constitute a major site of immune resistance (6–9). Functional interpretation requires multiplex spatial analysis, lineage-resolved marker assessment, and longitudinal clinical validation.

PD-1/PD-L1 blockade is now relevant across multiple UC settings. In BCG-unresponsive NMIBC with carcinoma *in situ*, pembrolizumab monotherapy demonstrated complete-response activity in KEYNOTE-057 among patients who were ineligible for or declined radical cystectomy (RC) (29). In advanced or metastatic UC, avelumab first-line maintenance after platinum-based chemotherapy improved overall survival in JAVELIN Bladder 100 (30). More recently, enfortumab vedotin plus pembrolizumab improved outcomes in previously untreated locally advanced or metastatic UC in EV-302/KEYNOTE-A39 (31–34). These trials provide important clinical context for UC immunotherapy and ADC-based therapy; however, none prospectively evaluated TLS as a predictive biomarker. Accordingly, any association between TLS status and benefit from these treatments remains correlative and hypothesis-generating. TLS-rich tumors should not automatically be considered ICI- or ADC-sensitive, and TLS-poor tumors should not automatically be excluded from these approaches.

## 5 Clinical decision points in urology: where TLS could be tested

From a urologist’s perspective, the clinical value of TLS will depend on whether it can improve decisions that are currently based mainly on stage, grade, carcinoma *in situ* status, lymphovascular invasion, variant histology, tumor size, multiplicity, completeness of resection, and response to BCG. In baseline TURBT and repeat TURBT, TLS assessment could be tested as an adjunct to conventional risk stratification, especially in high-grade T1 disease.

During BCG therapy, paired pre-BCG and post-induction TURBT or biopsy specimens could determine whether TLS maturation, persistence, inhibitory immune-cell enrichment, or absence correlates with response. In BCG-unresponsive disease, TLS status could be tested as an adjunctive biomarker for evaluating bladder-sparing systemic therapy, intravesical immune agonists, ADC-based combinations, or early RC.

At present, TLS cannot independently determine TURBT versus RC, continued intravesical therapy versus systemic therapy, or ICI/ADC treatment versus surgery. Its role is best viewed as a future adjunctive biomarker requiring prospective validation within guideline-defined risk categories.

## 6 Therapeutic contexts and future research on TLS-associated immune architecture

This section discusses therapeutic contexts in which TLS-associated immune architecture could be studied. At present, TLS remains an investigational biomarker and should not guide surgical decision-making or treatment selection.

### 6.1 TLS strategies in the era of antibody-drug conjugates

EV-associated tumor-cell injury has been described in preliminary conference data as having immunogenic-cell-death-associated features, including calreticulin exposure and release of damage-associated signals (35, 36). This preliminary observation provides a rationale for investigating local immune remodeling. However, it does not demonstrate that these events occur in UC patient tumors, that EV induces TLS neogenesis or maturation, or that TLS remodeling mediates clinical benefit from EV-based therapy. The clinical activity of EV plus pembrolizumab has been established in EV-302 (31), whereas the relationship between EV treatment and TLS architecture remains unknown.

### 6.2 STING agonist intravesical instillation: a potential strategy for local immune remodeling

Intravesical STING agonists represent a preclinical approach to local immune modulation in NMIBC. Experimental studies, including intravesical VB-85247 and conference-stage E7766 work, have reported local activation of type I interferon-related pathways and antitumor immune responses (37, 38). These studies do not demonstrate *de novo* TLS induction, conversion of immune-cold tumors, or bladder-preservation benefit in patients. Accordingly, STING agonists should be discussed as candidates for prospective trials in which TLS architecture is an exploratory translational endpoint rather than as a TLS-inducing therapeutic strategy.

### 6.3 TLS as a potential adjunct in surgical decision research

Whether standardized TLS assessment adds prognostic information beyond established clinicopathological criteria in high-grade T1 disease remains unknown. Retrospective UC studies have associated mature TLS-related immune features with favorable outcomes in selected cohorts (7–9). However, these observations do not establish that TLS maturity can identify patients who can safely continue bladder-preserving therapy or patients who should undergo early radical cystectomy.

TLS status may be investigated as an adjunctive variable in future high-grade T1 cohorts together with tumor grade, depth of invasion, carcinoma *in situ*, lymphovascular invasion, variant histology, multifocality, completeness of resection, and response to BCG. Low TLS maturity, TLS absence, or regulatory/inhibitory immune-cell enrichment should not be used independently to infer poor immunotherapy response, recommend early radical cystectomy, or defer guideline-based treatment. Prospective studies are required to determine whether standardized TLS scoring provides incremental value beyond established risk models.

Incorporating TLS maturity into risk stratification remains an investigational strategy. Future prospective studies should determine whether standardized TLS scoring can add clinically useful information beyond established clinicopathological models in high-grade T1 disease.

## 7 Methodological challenges and future perspectives in urological TLS research

As understanding of the key role of tertiary lymphoid structures (TLS) in tumor immunity deepens, their research value in urothelial carcinoma (UC) is becoming increasingly apparent. However, translating TLS from a biological discovery into a reliable clinical biomarker still faces a series of methodological challenges. This section aims to explore the key bottlenecks in current TLS research in urological oncology and to propose potential avenues for future breakthroughs.

### 7.1 From 2D IHC to 3D spatial transcriptomics: the challenge of standardizing TLS quantification in urological pathology

Traditionally, the identification and quantification of TLS have relied heavily on immunohistochemical (IHC) analysis of two-dimensional (2D) tissue sections, typically using markers such as CD20, CD3 and PNAd to localize B-cell zones, T-cell zones and neovascularization (39). However, this approach has clear limitations:

1. Information loss and bias: 2D sections cannot fully capture the three-dimensional spatial distribution of TLS within tumor tissue, their complete structure (such as germinal centers), or the precise distance from tumor cell boundaries, yet these spatial features are closely associated with prognosis (39).
2. Lack of standardization: There is currently no unified definition or scoring system for TLS. For example, what constitutes a ‘mature’ TLS? How should its density (number/area) or spatial distribution be quantified? The absence of such standards makes it difficult to compare and integrate results across different studies.

Beyond analytical platforms, TLS biomarker development is limited by the absence of a universally accepted scoring system defining TLS positivity, density, maturity, and spatial compartment. Studies variously rely on H&E morphology, CD20/CD3 aggregates, CD21/CD23-positive follicular dendritic cell networks, PNAd-positive HEVs, or germinal center markers such as BCL6 and Ki67. These differences make cross-study comparison difficult and limit reproducibility.

Spatial transcriptomics provides an approach for *in situ* analysis of gene expression while preserving spatial context, and multiplex immunohistochemistry (mIHC) can complement this readout by resolving cellular phenotypes (40). Together, these approaches can support the delineation of TLS and its microenvironment, including regions with high expression of CXCL13, CCL19, and CCL21 (15). They can also clarify spatial relationships among immune-cell subsets within TLS-like structures and their communication networks with tumor cells, while supporting investigation of candidate functional states such as germinal-center-like activity.

However, the widespread application of these advanced technologies in urological pathology still faces challenges, including high costs, complex data analysis workflows, and the requirement for high-quality fresh-frozen tissue. In the future, the development of optimized protocols suitable for formalin-fixed paraffin-embedded (FFPE) samples, alongside the establishment of open, standardized TLS spatial analysis workflows and databases, will be key to realizing the potential of TLS as a standardized biomarker.

Sampling is a particular issue in UC. TURBT specimens may capture only superficial lamina propria and may not adequately represent deeper stromal or invasive-front TLS. Intratumoral and inter-lesional heterogeneity can produce discordant TLS status across different tumor sites, especially in multifocal NMIBC. In routine pathology practice, distinguishing dense lymphoid aggregates from early or mature TLS may also show interobserver variability.

Importantly, BCG, cystitis, urinary tract infection, catheter irritation, hematuria, and recent TURBT can generate inflammatory lymphoid aggregates that mimic tumor-associated TLS. These confounders should be documented in future studies and considered when interpreting TLS in routine specimens.

### 7.2 Exploration of alternative markers for TLS in urine

Can urinary CXCL13 or APRIL levels be used for non-invasive monitoring of the TLS state in the bladder? Given the intraluminal growth characteristics of bladder cancer, urine serves as an ideal non-invasive source for obtaining tumor-related information. This raises a highly compelling research question: can the state of the TLS within the bladder be indirectly reflected by detecting specific molecules in urine?

CXCL13, as a key chemokine driving B-cell homing and TLS formation, is a prime candidate biomarker.

CXCL13 and APRIL are candidate urine biomarkers because they are linked conceptually to B-cell recruitment and survival. However, no direct UC study has established urinary CXCL13 or APRIL as a surrogate for tissue TLS density, maturity, or treatment response. The hypothesis that TLS- or immune-cell-derived CXCL13 may diffuse into urine therefore remains hypothesis-generating. Specificity is the major challenge: cystitis, urinary tract infection, catheter irritation, recent TURBT, hematuria, and BCG-induced granulomatous inflammation may all increase inflammatory chemokines or B-cell-related cytokines independently of tumor-associated TLS. Whether urinary concentrations quantitatively reflect the density, maturity, or functional status of TLS within a tumor, or predict treatment response or recurrence, remains unknown.

Technical validation: There is currently a lack of large-scale clinical studies systematically comparing the correlation between urinary biomarker levels and histological TLS characteristics (confirmed via IHC or spatio-omics).

Future research requires prospective cohorts with concurrent tumor-tissue and serial urine collection, standardized urine timing, and documentation of infection and instrumentation status. Such studies could test the feasibility and specificity of urinary CXCL13, APRIL, or other molecules as non-invasive research surrogates for tissue TLS; their clinical predictive value is unestablished.

Future studies should collect urine under standardized conditions, document infection and instrumentation status, include urine culture or inflammatory markers when appropriate, and correlate urinary protein or transcript levels with IHC/multiplex-confirmed TLS density and maturity.

### 7.3 UTUC: an underexplored setting for TLS research

Direct primary studies mapping TLS in upper tract urothelial carcinoma (UTUC) were not identified in the focused literature assessed for this review. UTUC should therefore be regarded as an important evidence gap rather than as a setting with established TLS biology or clinical implications.

Unique biological and clinical context: UTUC has anatomical, molecular, sampling, and management features that differ from bladder UC [general clinical background (41)]. A testable hypothesis is that TLS may be less frequent in UTUC but, when present, more prognostically informative. A second hypothesis is that Lynch syndrome-associated or MSI-high UTUC may show greater TLS density and more mature germinal-center features than sporadic UTUC. These questions could be tested first in nephroureterectomy cohorts using multiplex IHC panels for CD20, CD3, CD21/CD23, PNAd, BCL6, Ki67, FOXP3, and inhibitory immune-cell markers, because ureteroscopic biopsy tissue is limited. No direct human UTUC evidence currently establishes these hypotheses.

## 8 Conclusion

In the evolving landscape of urothelial carcinoma management beyond BCG failure, TLS provide a useful spatial framework for understanding local adaptive immunity and immune heterogeneity. In human UC cohorts, TLS maturity, density, and spatial localization have shown retrospective and correlative associations with immune context and clinical outcomes (6–9).

For urologists and translational researchers, TLS may be most valuable at present as an investigational adjunct rather than as a stand-alone clinical biomarker. TLS-informed profiling could be tested in future studies for high-risk T1 stratification, post-BCG immune assessment, selection of bladder-sparing clinical trial populations, and integration with tissue or urinary biomarkers. However, TLS status should not independently determine TURBT versus radical cystectomy, continuation of intravesical therapy, or transition to systemic therapy.

Future progress will require standardized TLS scoring systems, reproducible pathology workflows, paired longitudinal TURBT cohorts, spatial profiling, and tissue-validated urinary biomarkers. The next step is to determine whether TLS-informed immune profiling can improve risk stratification, inform clinical trial design, and support evidence-based bladder-preservation strategies without overextending TLS beyond its current level of validation.
